# Everybody's Page

**Published:** 1891-11-14

**Authors:** 


### EVERYBODY'S PAGE.

THE inability to remove from silver spoons the stain caused by eggs has often been a source of domestic despair. Both the despair and the stain can be removed, the latter by applying salt with a soft cloth.

---

A WRITER in *Temple Bar* has thrown cold water upon the popular fallacy (sic) in connection with fish, that the eating of that particular food increases brain power. We are not prepared to combat his assertion, that our fisher villages are not the seats of any great amount of brain power, but the fact that their views of life and its responsibilities are shrouded in a haze of superstition is not due to any lack of brain power owing to the consumption or non-consumption of fish, but to the circumstance that their inhabitants are uneducated and are constantly face to face with nature in one of her most awe-inspiring forms.

---

IT is a question where more object lessons in civility are to be gained: in a fashionable church or in the Metropolitan Railway during the hours when traffic is heaviest. As a couple in the humbler ranks of life were on the point of entering a compartment a few mornings ago, the better half, who, of course, was in the background, asked the partner of her joys and sorrows if there was room. "Only for one," said he, getting in, "you git elsewhere." It did not occur to this "nature's gentleman" to give *place aux dames*, being no doubt a firm believer in woman's rights, and her ability to look after herself. It is such amenities as this which, we presume, constitute the great charm of married life, the monotony of which they must help considerably to alleviate.

OWING to the great importance of having chloroform in an absolutely pure condition, chloroform prepared by crystallising out will probably be the only form in use ere long. It will be interesting to note whether in this form it will have any capricious effects on the heart.

---

ON first perusing the pages of Sir James Paget's "Studies of Old Case Books," the reader is struck by the fatal result of many surgical cases which in these days would hardly be considered as demanding more than ordinary care and skill on the part of the surgeon. Yet these fatal terminations, which would now be considered the result of gross incompetence, were not due to want of skill, but to the absence no doubt of the antiseptic treatment which has been of such value in modern surgery.

---

THE mosquito, which is the dread of all fresh comers into a country with which it is infested, and which has probably been productive of more unparliamentary, or perhaps, we should now say, parliamentary, language than any other insect, is not quite such a foe of mankind as he is usually painted. He has been pressed into a somewhat novel use by physicians for the purpose of conferring immunity against yellow fever, by causing healthy individuals to be stung by mosquitoes which have previously bitten those who have suffered from the fever. It is stated that experiments carried out for about ten years in this direction have met with such success as to show that this much-abused insect is a trustworthy attenuator of the yellow fever virus.

